# Fibroblast Activation Protein Compared with Other Markers of Activated Smooth Muscle Cells, Extracellular Matrix Turnover and Inflammation in a Mouse Model of Atherosclerosis

**DOI:** 10.3390/metabo15040243

**Published:** 2025-04-02

**Authors:** Adam Mohmand-Borkowski, Dareus O. Conover, Tomasz Rozmyslowicz

**Affiliations:** 1 Department of Cardiology, Cape Cod Hospital, Hyannis, MA 02601, USA; 2 School of Arts and Sciences, University of Pennsylvania, Philadelphia, PA 19104-6304, USA; dconover@upenn.edu; 3 Department of Pathology and Laboratory Medicine, University of Pennsylvania, Philadelphia, PA 19104, USA; rozmyslo@pennmedicine.upenn.edu

**Keywords:** fibroblast activation protein, vascular smooth muscle cells, atherosclerotic plaque

## Abstract

**Background:** Fibroblast activation protein (FAP) is a cell surface glycoprotein expressed by myofibroblastic cells in areas of active tissue remodeling, such as wound healing, fibrosis, and certain chronic inflammatory lesions. As FAP is uniquely present in chronic inflammatory lesions and has an important role in extracellular matrix (ECM) turnover, it appears to have all the characteristics necessary for involvement in atherosclerosis and atherosclerotic plaque rupture and has become a potential target in the treatment of myocardial infarction. **Methods:** To further understand the role of FAP, its expression in atherosclerotic plaques was examined in a genetically modified mouse model of accelerated atherosclerosis (*Apobec1* −/− *Ldlr* −/− double-knockout mice). The immunohistochemical Fap staining of atherosclerotic plaques in a mouse model of atherosclerosis was correlated with quantification of *Fap* mRNA obtained from the atherosclerotic plaques of the aortic arch. Fap distribution was characterized in mouse atherosclerotic plaques relative to other markers of activated smooth muscle cells, such as alpha smooth muscle actin and myosin heavy chain (Acta2 and Myh2), ECM turnover (Ki-67, procollagen III and Mmp-9), and inflammation in atherosclerosis (Cd-44, Il-12 and Tgf beta) using immunohistochemistry (IH) and RT-PCR analysis. **Results:** The mouse model of accelerated atherosclerosis showed an increasing presence of Fap with the progression of atherosclerosis and a high expression level in advanced atherosclerotic lesions compared with other markers of ECM turnover and inflammation in atherosclerosis. **Conclusions:** FAP exhibits a distinct pattern of expression in a mouse model of atherosclerosis as compared to other markers of activated vascular smooth muscle cells, ECM degeneration, and inflammatory cytokines.

## 1. Introduction

Atherosclerotic plaque rupture and, less frequently, plaque erosion are the most common mechanisms of coronary events. Atherosclerotic plaque rupture causes acute thrombosis in the place of rupture and, subsequently, the occlusion of coronary vessels. This leads to myocardial infarction, a fatal complication of atherosclerosis. Significant efforts have been devoted to characterizing and detecting plaques prone to rupturing (“vulnerable plaques”) due to their significant impact on morbidity and mortality [1,2,3,4,5]. Extracellular matrix (ECM) turnover is a critical process for atherosclerotic plaque progression and rupture; however, the exact mechanism of this process is not well defined. It has been shown that high contents of vascular smooth muscle cells (VSMCs) and collagen, a thick fibrous cap, and a lack of inflammation are determinants of plaque stability. Ongoing inflammation and a paucity of fibrosis make plaques more prone to rupture with subsequent clinical coronary events. With the balance between inflammation and fibrosis being critical for plaque stability, a better understanding of VSMC/myofibroblast proliferation and signaling in atherosclerotic plaques is necessary to develop preventive therapies for acute coronary syndromes [6,7,8,9,10,11,12,13,14].

Fibroblast Activation Protein (FAP) is a cell surface glycoprotein expressed exclusively by myofibroblastic cells in areas of active tissue remodeling, such as wound healing, fibrosis, or chronic inflammatory lesions, but it is mostly absent in normal tissues. FAP has both dipeptidyl peptidase and collagenase activity, and is capable of degenerating gelatin and collagen II, leading to ECM degradation and the activation of growth factor [15,16,17]. The loss of FAP expression can also upregulate compensatory pathways, such as metalloproteinase (MMP) activation [18]. Tissue distribution suggests that FAP is a part of a smooth muscle cell’s proliferation and differentiation program with an expression pattern that is different from those of other markers of activated VSMCs, such as alpha smooth muscle actin.

The presence and increased expression of FAP in the progression of human atherosclerotic plaques have been shown in previous studies, although it has not been seen to be expressed in the coronary vessels without atherosclerosis [19,20,21,22]. It has been postulated that FAP may play a critical role in the development of atherosclerotic plaques by modulating the interaction between inflammatory response and fibrotic remodeling. However, the role of FAP expression by smooth muscle cells in atherosclerotic plaques and in plaque rupture, resulting in acute coronary syndromes, are not well understood.

The role of FAP in atherosclerosis has been studied using a murine model of accelerated atherosclerosis (*Apoe* −/− *and Ldlr* −/− mice) and a genetic approach with *Fap* −/− knockout mice [23,24]. Previous studies supported the role of FAP in the fibroinflammatory process of atherosclerosis, with Fap mostly being expressed on the surface of vascular smooth muscle cells in murine atherosclerotic lesions. Furthermore, *Fap* deletion decreased the progression of atherosclerosis in an experimental mouse model [23].

To further characterize the mechanism and role of FAP in ECM remodeling and the inflammatory process of atherosclerosis, we compared the expression levels of Fap in atherosclerotic plaques from *Apobec1* −/− *Ldlr* −/− double-knockout −/− mice to other markers of activated vascular smooth muscle cells, ECM turnover and inflammation, modulating the process of atherosclerosis. Subsequently, immunohistochemical Fap staining of atherosclerotic plaques in ascending aorta from *Apobec-/-ldlr* −/− mice was correlated with an analysis of the genetic material obtained from atherosclerotic plaques.

## 2. Materials and Methods

### 2.1. Animal Model

To establish the presence and distribution of Fap in the mouse model of atherosclerosis and atherosclerotic plaques, 15 *Apobec1* −/− *Ldlr* −/− mice were used in the experiment, including five 13-week-old males (no significant atherosclerosis), five 23-week-old males (moderate atherosclerosis) and five 33-week-old males (advanced atherosclerosis). Because mice, unlike humans, less frequently develop atherosclerosis in the coronary arteries, but readily develop atherosclerosis in the proximal aorta, the aortic root and proximal aorta were used in the study [25,26]. The process for the acquisition and use of animal samples was reviewed and approved by the Wistar Institute IACUC/Letter-1/12.18.2020.

### 2.2. Tissue Preparation

Mice aorta sections were obtained using a previously described protocol [25] and embedded in the OCT medium. Following this, 10 mm cross-sections of the aortic root, arch and thoracic aorta (used as the control) were cut and placed on microscope slides. To analyze atherosclerotic lesion burden, equally spaced sections were stained with hematoxylin-2 and eosin-Y (Thermo Fisher Scientific, Waltham, MA, USA) according to the manufacturer’s protocols. Aortic arch tissue (5 mice in each group) was used to analyze the quantity of *Fap* mRNA and the mRNA transcribed from other genes examined in this study. In the group with 33-week-old mice, thoracic aortas were also taken for RNA analysis as a control for the aortic root/arch samples. The aortic root and proximal aorta were used for immunochemical staining after prior conservation in an OCT compound.

### 2.3. mRNA Isolation

For mRNA isolation, aortic segments were immediately cut into small pieces and lysed in 350 µL of TRIzol reagent (Invitrogen, Carlsbad, CA, USA). After the homogenization and lysis of aortic tissue, an RNAeasy kit (Qiagen, Hilden, Germany) was used for the fast purification of high-quality RNA, which was then collected in 25 µL of RNase-free water. TaqMan quantitative reverse transcriptase RT-PCR was used to quantify the mRNA levels for selected genes.

### 2.4. PCR Analysis

Quantitative PCR (qPCR) was performed on an ABI Instrument (Applied Biosystems, Foster City, CA, USA) with real-time fluorescence to measure the quantity of DNA. A fluorescent signal was generated with a double-stranded DNA-binding dye (SYBRGreen). Quantification cycle values were used to evaluate the relative target abundance between all sample groups (13-, 23- and 33-week-old *Apobec1* −/− *Ldlr* −/− double-knockout mice). It allowed for the sensitive and specific detection and quantification of *FAP* mRNA and mRNAs of other markers, characterizing atherosclerotic plaque with a specific primer sequence in the polymerase chain reaction (PCR) for *Fap*, *Acta2*, *Myh2*, *Tgf β*, *Ki-67*, *Col3a1*, *Mmp9*, *Cd44* and *Il-12.* Each reaction had a final volume of 30 μL, a template of 0.1ng/μL, and a primer concentration of 500 nM each.

### 2.5. Immunohistochemistry

Immunohistochemistry (IH) was performed on proximal mouse aorta with primary anti-mouse antibodies against mouse Fap protein for a comparison with other markers of activated vascular smooth muscle cells, inflammation, and proliferating cells. For staining, we applied rat anti-mouse Ki-67 and rat anti-mouse CD-44 antibodies (Invitrogen, Carlsbad, CA, USA), rabbit anti-mouse Fap and rabbit anti-mouse Myh2 antibodies (Fisher Scientific, Pittsburg, PA, USA), mouse anti-mouse Acta2 antibody (Invitrogen, Carlsbad, CA, USA), followed by appropriate biotinylated secondary antibodies (Sigma-Aldrich, St. Louis, MO, USA) for the amplification of signals in IH and direct visualization. Imaging of histological sections was performed using an Olympus BH-2 microscope with an Olympus DP-10 camera.

### 2.6. Correlation Between Immunochemistry and Gene Expression

The correlation between the immunohistochemical Fap staining of atherosclerotic plaques in the aortic root of the mouse model (*Apobec1* −/− *Ldlr* −/− double knockout) and an analysis of genetic material obtained from atherosclerotic plaques of the aortic arch was performed. Following this, the characterization o of the *Fap* expressionin mouse model of atherosclerosis was conducted in comparison with other markers of activated smooth muscle cells, such as alpha smooth muscle actin and myosin heavy chain (*Acta2* and *Myh2*), ECM turnover (*Ki-67, type III procollagen* and *Mmp-9),* and inflammation in atherosclerosis *(Cd-44, Il-12* and *Tgf beta).*

### 2.7. Statistical Analysis

The data are presented as the mean ± standard deviation. The arithmetic means and standard deviations were determined using Microsoft Excel 16.93.1.

## 3. Results

### 3.1. Fap Expression in Atherosclerotic Lesions

**Figure 1:**
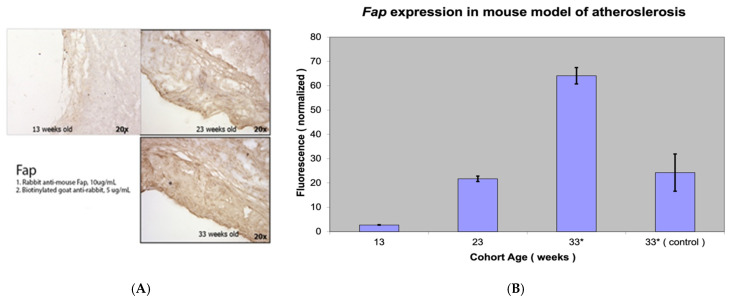
Fap staining (**A**) and *Fap* expression (**B**) in a mouse model of atherosclerosis (apo(*Apobec1* −/− *Ldlr* −/− knockout mice)bStatistically highly significant *p* value < 0.001.

Immunohistochemical staining of atherosclerotic lesions in *Apobec1* −/− *Ldlr* −/− double-knockout mice showed minimal Fap staining in the pre-atherosclerotic group (13-week-old mice), positive Fap staining in the early atherosclerotic group (23-week-old mice) and pronounced Fap staining in the advanced atherosclerotic group (Figure 1).

RT-PCR with real-time fluorescence measuring relative F*ap* *mR*NA abundance between sample groups (13-, 23-, and 33-week-old Apob*ec1 −/− Ldlr −/− dou*ble-knockout mice) showed minimal F*ap* *mR*NA levels in the proximal aorta in the 13- and 23-week-old mice and high levels of F*ap* *mR*NA in the 33-week-old mice with advanced atherosclerosis. The difference in FAP *Fap*ession was highly statistically significant (*p* value < 0.001). The sample specimen (used as a control) from the more distal, thoracic aorta of the 33-week-old mice showed the presence of FAP, *ap* expected. However, it was less pronounced than the samples from the aortic root as the aortic root and arch are most prone to atherosclerotic progression in a mouse model of atherosclerosis (Figure 1).

### 3.2. Comparison of Fap Expression with Other Markers of Activated Vascular Smooth Muscle Cells

**Figure 2:**
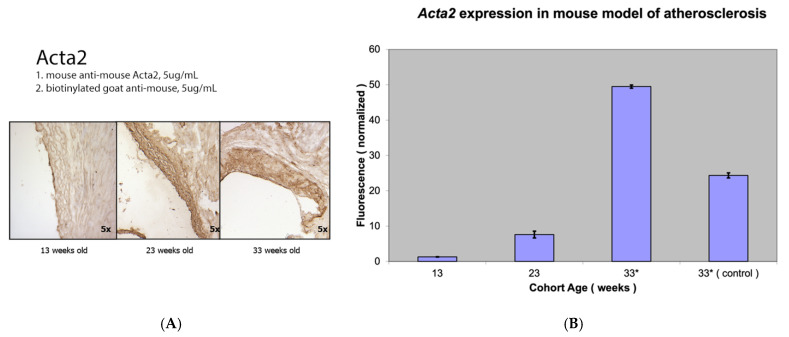
Alpha smooth muscle actin (Acta2) staining (**A**) and *Acta2* expression (**B**) in a mouse model of atherosclerosis (*Apobec1* −/− *Ldlr* −/− knockout mice). * Statistically highly significant *p* value < 0.001.

**Figure 3:**
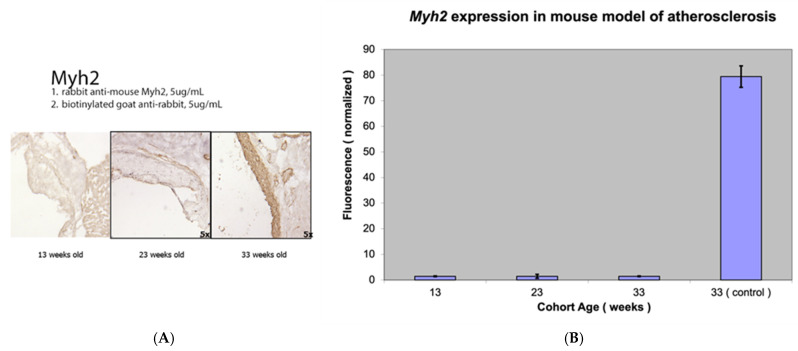
Myosin heavy chain (Myh2) staining (**A**) and *Myh2* expression (**B**) in a mouse model of atherosclerosis (*Apobec1* −/− *Ldlr* −/− double-knockout mice).

**Figure 4:**
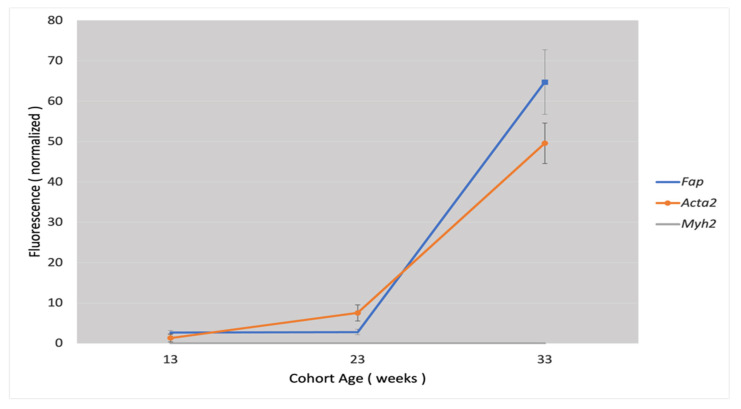
Graphical comparison of *Fap* expression versus the expression of other markers of vascular smooth muscle cells in a mouse model of atherosclerosis, depending on progression of atherosclerotic lesions.

The expression levels of *Fap*, a marker of activated smooth muscle cells, were compared with those of alpha smooth muscle actin (a marker for the contractile, pro-fibrogenic phenotype of VSMCs) and myosin heavy chain (a marker of activated contractile myofibroblasts) (Figure 2, Figure 3 and Figure 4).

Similar increases in *Fap* and *Acta2* mRNA levels were observed with the progression of atherosclerosis. Differences in *Acta2* expression between the three groups were again highly statistically significant (*p* value < 0.001). Interestingly, with relatively limited but visible Myh2 staining using immunocytochemistry in atherosclerotic lesions, no significant *Myh2* mRNA levels were detected using RT-PCR from the proximal aorta of the mouse model of atherosclerosis compared to the abundant *Myh2* mRNA levels from the thoracic aorta (Figure 3).

Figure 4 presents a direct graphic comparison of *Fap* expression with the expressions of other markers of vascular smooth muscle cells *(Acta2* and *Myh2)* depending on the progression of atherosclerotic lesions in the mouse model.

### 3.3. Fap Expression Versus Expression of Markers of ECM Turnover

**Figure 5:**
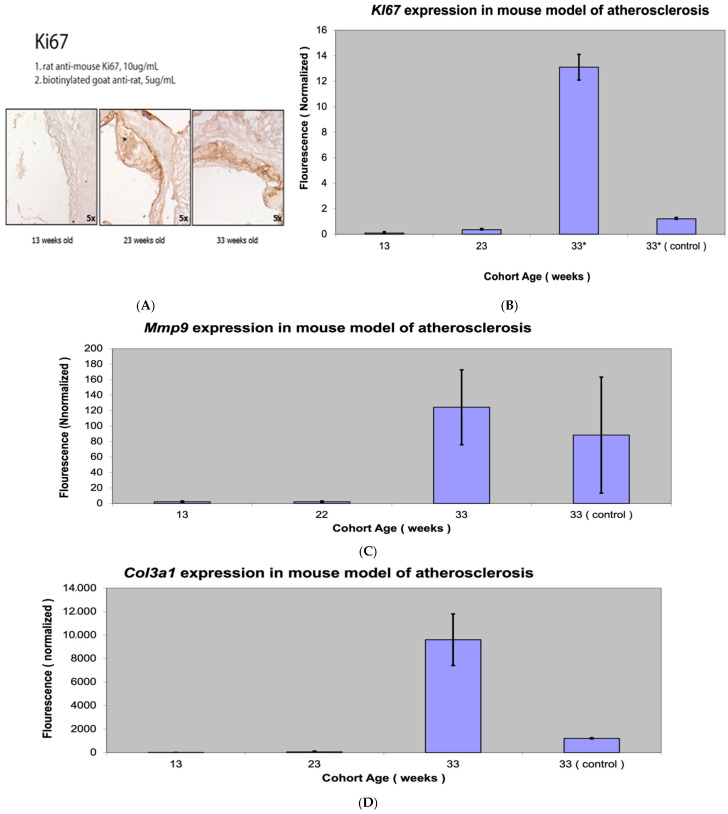
Ki-67 staining (**A**) and *Ki-67* expression (**B**), *MMP-9mp*xpression (**C**), and procollagen III *(Col3a1)* expression (**D**) in a mouse model of atherosclerosis (*Apobec1* −/− *Ldlr* −/− double-knockout mice). * Statistically highly significant *p* value < 0.001.

The expression of three markers of ECM turnover was compared to *Fap* expression in atherosclerotic plaques: *Ki-67,* a marker of active cell division in different stages of atherosclerosis; procollagen type III *(Col3a1),* a marker of collagen synthesis and degradation in ECM; and metalloproteinases 9 *(Mmp-9),* which is induced by inflammation in atherosclerosis with higher levels of *Mmp-9* in plaques favoring ECM destruction (Figure 5).

As expected, all markers of ECM degeneration—*Ki-67*, *Col3a1,* and *Mmp-9*—had the highest expression in mice with advanced atherosclerosis (33-week-old group). Differences in *Ki-67* and *Col3a1* expression between the three groups was again highly statistically significant (*p* value < 0.001). There was no *Mmp9* expression in the 13- and 23-week-old *Apobec1* −/− *Ldlr* −/− mice, but high *Mmp-9* expression was present in the 33-week-old group (advanced atherosclerosis lesions).

**Figure 6:**
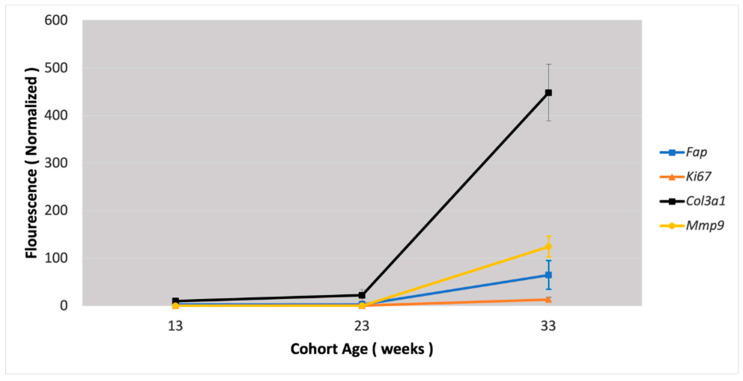
*Fap* expression versus expression of ECM turnover markers in mouse model of atherosclerosis depending on progression of atherosclerotic lesions.

To correlate the results of RT-PCR with IH, we performed Ki-67 staining of atherosclerotic lesions, with consistent results showing increased staining and expression of other inflammatory markers with the progression of atherosclerosis (Figure 5). The *Fap* mRNA expression curve followed the *Mmp-9* and *Ki-67* curves with the progression of atherosclerosis, with procollagen showing a markedly higher presence in advanced atherosclerotic lesions (Figure 6).

### 3.4. Fap Expression Against Inflammation Markers in Atherosclerotic Lesions

The expression of the following inflammatory markers in atherosclerotic plaques was tested against *Fap* expression: *Tgf beta*—a stimulator of collagen synthesis and a regulator of the inflammation process and fibrosis; *Il-12*—a pro-inflammatory cytokine expressing pro-atherosclerotic function; and *Cd44*—a marker of activated lymphocytes and vascular cells regulating leukocyte recruitment in inflammatory sites.

**Figure 7:**
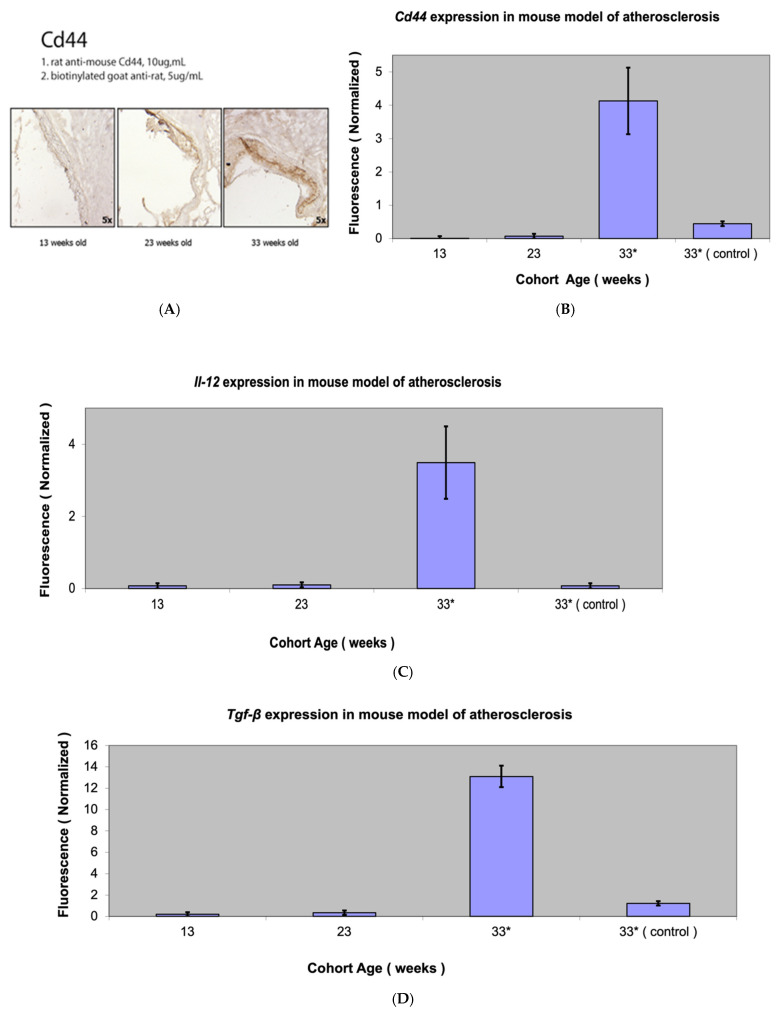
Cd44 staining (**A**) and *Cd44* expression (**B**), *Il-12* expression (**C**), and *Tgf beta* expression (**D**) in mouse model of atherosclerosis (*Apobec1* −/− *Ldlr* −/− double-knockout mice). * Statistically highly significant *p* value < 0.001.

The expression of all inflammatory markers showed an acute increase in mice with marked atherosclerosis (33-week-old group). Differences in the *Cd-44*, *Il-12*, and *Tgf beta* expression between the three groups were highly statistically significant (*p* < 0.001). The results of the gene expression analysis were validated with immunohistochemical Cd-44 staining of atherosclerotic lesions, which showed increased staining with the progression of atherosclerosis (Figure 7).

**Figure 8:**
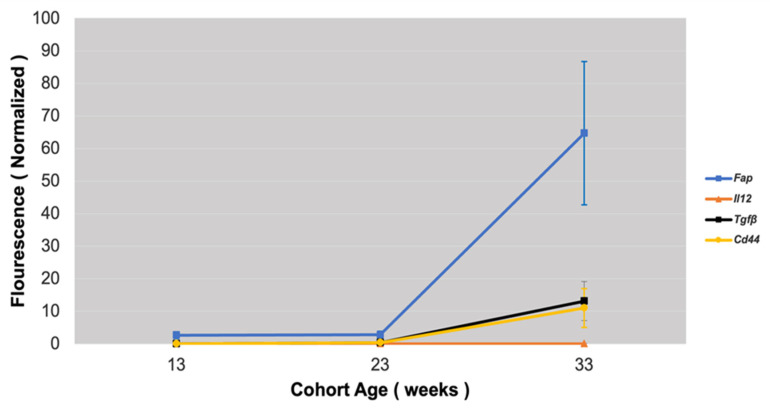
Graphical comparison of *Fap* expression versus inflammatory molecules in mouse model of atherosclerosis depending on progression of atherosclerotic lesions.

*Fap* mRNA expression followed increases in the expression of inflammatory markers with the progression of atherosclerosis, with a relatively higher presence of *Fap* mRNA levels being found in advanced atherosclerotic lesions (Figure 8).

## 4. Discussion

The genetically created mouse model of atherosclerosis provides opportunities to better understand the in vivo role of FAP in atherosclerosis and examine its expression in the atherosclerotic lesions of *Apobec1* −/− *Ldlr* −/− double-knockout mice. Immunohistochemical staining and an analysis of the genetic material showed no significant Fap expression in early atherosclerosis with a marked presence of Fap in advanced atherosclerotic lesions. Fap appears to have a distinct expression pattern compared to other markers of activated VSMCs (Acta2 and Myh2) and proliferating or inflammatory molecules in mice models of atherosclerosis. The results of IH staining are consistent with the analysis results of the genetic material from atherosclerotic lesions. The increased Fap expression in advanced atherosclerotic lesions is consistent with other studies on murine models of atherosclerosis using *Apoe* −/− and *Ldlr* −/− mice [23,24]. Although *Fap* deletion was shown to decrease the progression of atherosclerosis and increase the fibrillar collagen content in the murine model of atherosclerosis, the exact role of FAP in atherosclerosis is not fully understood.

In our study, Fap expression acutely rises in advanced atherosclerotic plaques and closely follows the expression of ECM degeneration and local markers of inflammation. The increased Fap expression in advanced atherosclerotic lesions compared to other markers of inflammation and ECM turnover suggest Fap’s important role in the balance between inflammation and fibrosis processes in atherosclerosis. Studies using PET imaging with FAP inhibitors to further characterize its role in murine atherosclerotic plaques can improve our understanding of this process [27,28,29].

Growing evidence that FAP regulates atherosclerosis by modulating the inflammatory response involved in plaque rupture and fibrosis supports the role of FAP in mediating plaque stability and susceptibility to acute coronary events. The complexity of FAP’s role in atherosclerotic plaques has been shown in a study linking FAP uptake in the arterial wall with marked calcification and calcified plaque burden but not the presence of established cardiovascular risk factors [30]. Additionally, although most vascular FAP expression can be attributed to locally activated fibroblasts, the origin of circulating FAP is not fully understood. Studies on systemic FAP concentrations during atherosclerosis-related ischemic events showed that a circulating FAP concentration does not correlate with FAP expression in tissues assessed by molecular imaging during acute ischemic events [31,32,33]. A decline in systemic FAP concentrations was noted acutely after myocardial infarction, with the maximum C-reactive protein level independently being associated with a low FAP concentration. These results are intriguing, as in our study, a local increase in FAP expression in a mouse model of atherosclerosis is associated with increased markers of inflammation in the atherosclerotic plaque, suggesting that local FAP expression is likely related to local fibroblast activation. The role of FAP in acute coronary syndromes may exceed the impact on the ECM and plaque stability as fibrinolysis inhibitor α2-antiplasmin was found to be a potential substrate of soluble FAP [34]. This may implicate FAP in thrombosis, complicating atherosclerotic plaque rupture. Ongoing attempts with immune-based therapeutic interventions targeting FAP in areas of active tissue remodeling suggest that specific interventions aiming at subpopulations of VSMCs expressing FAP in coronary disease may be useful in the future, and experimental models are currently being developed [23]. Additionally, the potential role of FAP in coronary disease is exemplified by the results of studies using FAP radiotracers with PET and SPECT imaging. These studies indicate that FAP signaling may be a novel biomarker of fibroblast activation after myocardial infarction, which is complimentary to traditional imaging and may be used for non-invasive evaluation of fibroblastic activation in atherosclerosis [35,36,37].

It has also been shown that after acute myocardial infarction, FAP upregulation occurs in the infarct region and attached myocardium. FAP may also play a distinct role in replacing fibrosis in the scar, and reactive fibrosis in non-infarcted myocardium [38]. The role of FAP as a diagnostic and therapeutic target in coronary artery disease is broadened to subsequent ischemic cardiomyopathy and heart failure. Potential applications of FAP can include the identification of early fibrosis in cardiomyopathy, the assessment of therapeutic responses and the prediction of cardiac dysfunction.

With very broad potential applications of FAP in clinical medicine in the near future, further research using FAP radiotracers will allow for better understanding of fibroblast activation in acute coronary syndromes and healing responses after myocardial infarction. Additionally, progress in the development of specific FAP inhibitors will help determine the diagnostic and therapeutic roles of FAP in cardiovascular medicine.

### Study Limitations

Although *Apobec1* −/− *Ldlr* −/− double-knockout mice represent well-established models of atherosclerosis, they exhibit distinct differences to human atherosclerosis, such as a paucity of unstable plaques and different anatomical sites of atherosclerotic lesions. Additionally, the relatively small sample size and the exclusive use of male mice, which did not account for sex differences in atherosclerotic plaque content, represent other limitations of this study.

## 5. Conclusions

Fap exhibits a distinct pattern of expression in a mouse model of atherosclerosis as compared to other markers of activated VSMCs, ECM degeneration, and inflammatory cytokines.

## Data Availability

The original contributions presented in this study are included in the article. Further inquiries can be directed to the corresponding author.
